# Comparison of STONE Score, Guy’s Stone Score, CROES Nomogram, and Seoul National University Renal Stone Complexity Score in Prognosticating Outcomes of Multiple-Tract Mini-Percutaneous Nephrolithotomy: A Retrospective Study

**DOI:** 10.7759/cureus.54790

**Published:** 2024-02-23

**Authors:** Khalid Farooq, Najma Hameed, Zainab Zaib, Muhammad Bilal Hameed, Husnain Ausaf, Fraz Shakil, Muhammad Afzaal Nawaz

**Affiliations:** 1 Urology, Lady Reading Hospital, Peshawar, PAK; 2 Radiology, Northwest General Hospital, Peshawar, PAK; 3 Obstetrics and Gynecology, Lady Reading Hospital, Peshawar, PAK; 4 Urology, Institute of Kidney Diseases, Peshawar, PAK; 5 Urology, Wexham Park Hospital, Slough, GBR; 6 Ophthalmology, Mayo Hospital, Lahore, PAK; 7 Urology, Muhammad Ali Hospital, Lahore, PAK

**Keywords:** renal complications, clinical research office of the endourological society (croes), stone free rate, renal stone complexity score, guy's score, stone score, multi-tract mini pcnl

## Abstract

Objective

The objective of this study was to compare the STONE score (Size of the stone, Topography or location, degree of Obstruction of the urinary system, Number of stones, and Evaluation of Hounsfield units), Guy’s stone score (GSS), Clinical Research Office of the Endourological Society (CROES) nomogram, and Seoul National University Renal Stone Complexity Score (RSCS) in prognosticating multiple tract mini-percutaneous nephrolithotomy (mPCNL) outcome.

Methodology

This descriptive retrospective analysis was carried out at the Urology Department, Lady Reading Hospital, Peshawar, Pakistan. Male and female patients in the age range of 18-70 years who underwent multiple tract mPCNL for renal stones from July 1, 2021, to June 30, 2023, were included in the analysis.

Results

A total of 110 patients were registered. Stone-free status (SFS) was achieved in 78.2% (n=86), and complications were observed in 13.6% (n=15) patients. The odds ratio for STONE score, GSS, CROES scoring system, and RSCS for predicting the SFS was 7.093 (95%CI 2.40-20.89), 9.333 (95%CI 2.92-29.81), 11.70 (95%CI 2.56-53.38) and 3.450 (95%CI 1.25-9.53), respectively.

Conclusion

Multiple tract mPCNL is a safe and effective technique for the management of renal stones, producing a high stone-free rate. This study demonstrated adequate efficacy and dependability of the four scoring systems in predicting SFS.

## Introduction

The history of renal stones traces back to 4000 B.C. [1]. The development of renal stones primarily occurs in the kidneys. It may dislodge and trap anywhere down the urinary tract; however, the most common site of impaction is within the kidneys [2]. Renal stone prevention remains an important concern in the discipline of urology. The clinical spectrum of renal stones is vast. The symptoms range from asymptomatic stones to renal or ureteric colic, hematuria, and obstruction to urinary flow to secondary infection leading to urosepsis and chronic kidney disease in long-term sequela [3,4].

The tools to manage renal stones have drastically evolved over the years. Percutaneous nephrolithotomy (PCNL) is a minimally invasive technique for renal stone extraction [5]. It has been subjected to several modifications like mini-PCNL (mPCNL) [6]. These adjustments and modifications have been implemented to reduce morbidity, analgesic needs, and hospital stay duration [7].

Despite these improvisations, none of the techniques have been completely effective and risk-free because the outcomes of the procedures depend on several factors [8]. Several scoring systems have been devised that take into account various factors for predicting the effectiveness and complications of these techniques. STONE (Size of the stone, Topography or location, degree of Obstruction of the urinary system, Number of stones, and Evaluation of Hounsfield units) score, Guy’s stone score (GSS), CROES (Clinical Research Office of Endourological Society) nomogram, and Seoul National University Renal Stone Complexity Score (RSCS) are among such scores [9]. This study aimed to compare the performance of STONE score, GSS, CROES nomogram, and Renal Stone Complexity scores in prognosticating the outcome of multiple-tract mPCNL. 

## Materials and methods

This descriptive retrospective study was carried out at the Urology Department, Lady Reading Hospital, Peshawar, Pakistan. Male and female patients aged 18-70 years who underwent multiple-tract mPCNL for renal stones from July 1, 2021, to June 30, 2023, were analyzed. The study was approved by the Lady Reading Hospital Institution Review Board (approval number: 28/LRH/MTI). Patients with incomplete medical records were excluded from the study.

A total of 110 patients were included in this study. The sample size was calculated using the WHO sample size calculator, taking an anticipated proportion of effectiveness of mPCNL as 76.0% with an 8% margin of error and 95% confidence level [10]. 

PCNL was performed in prone position using sheath sizing ≤18 French (Fr); utilizing two or more accessing tracts was called multiple-tract mPCNL. The outcome was in terms of the effectiveness of the procedure. Effectiveness was measured in terms of achievement of stone-free status (SFS) using non-contrast-enhanced computed tomography of kidneys, ureters, and bladder (CT KUB) performed at the end of the fourth post-operative week. Absence or stone fragments <4mm were recognized as SFS. Post-operative complications occurring within four weeks after surgery were evaluated using the Modified Clavien Classification System (MCCS) and grouped as low and high grade. Patients with none or MCCS grade I and II complications were called low grade, and those with MCCS grade III to V were called high grade. 

Relevant information and details were retrieved from the hospital management information system. Baseline parameters of interest and recorded pre-operative data, including patients' age, gender, body mass index (BMI), comorbidities like diabetes and hypertension, prior surgical history, and pre-operative examination findings, were retrieved. Pre-operative routine investigations included a complete blood profile, viral profile, coagulation profile, urinalysis, and renal functions. Pre-operative non-contrast-enhanced CT KUB was evaluated to draw stone characteristics like stone localization, size, and burden. Operative characteristics included duration of surgery, number of tracts, and entry points into the collecting system. All procedures were carried out under general anesthesia in a prone position after ureteral catheterization in the lithotomy position. A fluoroscopic-guided 18-G Chiba needle was employed to obtain entry into the pelvicalyceal network. A guidewire was introduced, and the tract was dilated with Amplatz dilators.

The disintegration of the stone was accomplished using a pneumatic lithotripter and extricated with graspers. Another tract was created similarly for stones at distant locations that cannot be retrieved with prior tracts. Standard post-operative care was given. Patients were discharged once stable. Length of hospital stay was recorded. Follow-up evaluation was carried out at the end of the fourth post-operative week with non-contrast-enhanced CT KUB for stone-free clearance and clinical evaluation for any complications. STONE score, GSS, CROES nomogram, and RSCS were calculated for each patient. The performance of each scoring tool for predicting the effectiveness was determined.

Data analysis

Data was analyzed using IBM SPSS Statistics for Windows, Version 24.0 (Released 2016; IBM Corp., Armonk, New York, United States). Continuous data was presented as mean±SD or median (interquartile range (IQR)). The normality of the data was checked with the Shapiro-Wilk test. Frequencies and percentages were computed for categorical data. As appropriate, continuous data was compared using the Student's t-test or the Mann-Whitney U test. Categorical data was compared using the chi-square test or Fischer exact test. Receiver operating curve (ROC) analysis was utilized to assess the predictive performance of the scoring systems for outcomes. Sensitivity and specificity were determined for scoring tools for predicting effectiveness. A contingency table analysis was carried out to measure the association. The odds ratio and Cramer v values were determined to assess the strength of the association. The cut-off for statistical significance was set at p-value ≤ 0.05.

## Results

Out of the total 110 patients, 86 (78.2%) achieved SFS (SFS group), and the remaining 24 patients (21.8%) failed to achieve SFS (non-SFS group). The mean age of the patients in the SFS group was 32.90±10.98 compared to 36.54±12.19 in the non-SFS group. The number of male participants in SFS and non-SFS groups was 59 (58.6%) and 15 (62.5%), respectively. Suitable kidney stones were more common than left, 45 (52.3%) versus 13 (54.2%) in SFS achieved versus non-SFS groups, respectively. Minor obstructive changes were observed in 68 (79.1%) patients in the SFS group and 20 (83.3%) in the non-SFS group. Both groups were significantly different regarding mean STONE score, GSS, CROES nomogram, and RSCS with p-values < 0.05, as shown in Table 1.

**Table 1:** Comparison of baseline and pre-operative characteristics between the group that achieved SFS and the group that did not achieve SFS BMI: body mass index; SFS: stone-free status; STONE: Size of the stone, Topography or location, degree of Obstruction of the urinary system, Number of stones, and Evaluation of Hounsfield units; CROES: Clinical Research Office of the Endourological Society; RSCS: Seoul National University Renal Stone Complexity Score

Variables	Sub-Groups	SFS Achieved (n = 86)	SFS Not Achieved (n = 24)	p-value
Age (years), mean ± SD		32.90±10.980	36.54±12.190	0.163
BMI (kg/m^2^), mean ± SD		24.68±2.519	24.19±2.37	0.400
Gender, n (%)	Male	59 (58.6%)	15 (62.5%)	0.573
	Female	27(31.4%)	09(37.5%)	
Stone size (mm), mean ± SD		16.28±2.51	18.42±5.32	0.108
Stone Number, n (%)	Single	34 (39.5%)	14 (58.3%)	0.129
	Multiple	30 (34.9%)	08 (33.3%)	
	Staghorn	22 (25.6%)	02 (8.3%)	
Side, n (%)	Right	45 (52.3%)	13 (54.2%	0.873
	Left	41 (47.7%)	11 (45.8%)	
Hydronephrosis, n (%)	None/Mild	68 (79.1%)	20 (83.3%)	0.644
	Moderate/ Severe	18 (20.9%)	04 (16.7%)	
Scores, mean ± SD	STONE sore	9.47±2.46	9.08±2.08	0.000
	Guy’s Stone Score	2.19±1.11	3.25±0.737	0.000
	CROES nomogram score	226.81±79.981	337.42±65.583	0.000
	RSCS	4.30±2.29	5.83±1.85	0.003

Table 2 shows that the mean operative time was 48.42±8.33 minutes in the non-SFS group and 44.70±6.52 minutes in the SFS group. The p-value for the difference in operative time was 0.022, which is less than 0.05; hence, it was statistically significant. However, there was no statistically significant difference in both groups in terms of number of tracts utilized (p-value>0.05).

**Table 2:** Comparison of operative characteristics between group that achieved SFS and the group that did not achieve SFS SFS: stone-free status

Variables	Sub-groups	SFS Achieved (n = 86)	SFS Not Achieved (n = 24)	p-value
Operative time (minutes), mean ± SD)		44.70±6.52	48.42±8.33	0.022
Number of tracts, n (%)	2	70 (81.4%)	16 (66.7%)	0.122
	>2	16 (18.6%)	08 (33.3%)	

The mean length of hospital stay was significantly higher in the non-SFS group (p-value <0.05). A statistically significant difference was observed in the rate of complications in both groups, with eight (33.3%) patients with high-grade complications in the non-SFS group in contrast to seven (8.1%) in the SFS group with a p-value of 0.001, as shown in Table 3.

**Table 3:** Comparison of post-operative characteristics between the group that achieved SFS and the group that did not achieve SFS Modified Clavien System (MCCS) was used for grading of complications. Patients with none or MCCS grade I and II complications were called low grade, and MCCS grade III to V were called high grade. SFS: stone-free status

Variables	Sub-groups	SFS Achieved (n = 86)	SFS Not Achieved (n = 24)	p-value
Length of hospital stay (days), mean ± SD		3.37±1.074	5.37±1.209	0.000
Complications, n(%)	None/low grade	79 (91.9%)	16 (66.7%)	0.001
	High grade	7 (8.1%)	8 (33.3%)	

**Figure 1:**
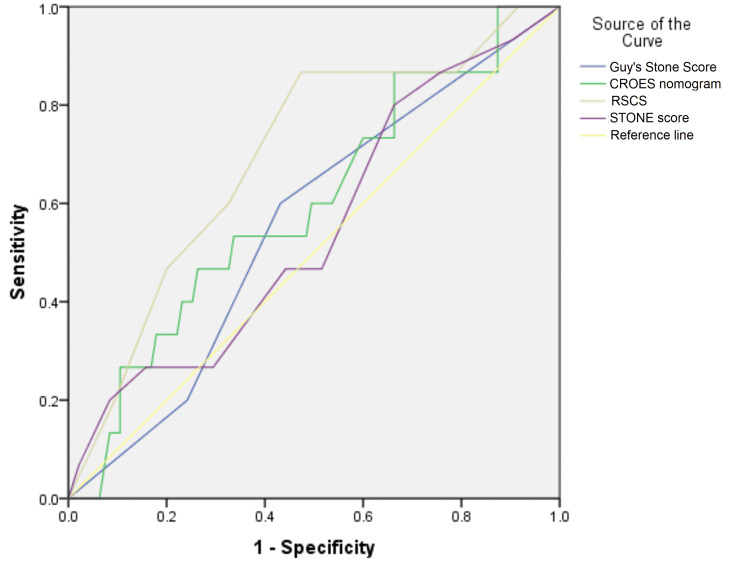
ROC curve of STONE score, CROES nomogram, Guy’s stone score, and RSCS for predicting the effectiveness of multiple tract mini-PCNL for renal stones y-axis: sensitivity; x-axis: 1-specificity; Diagonal segment are produced by ties STONE: Size of the stone, Topography or location, degree of Obstruction of the urinary system, Number of stones, and Evaluation of Hounsfield units; CROES: Clinical Research Office of the Endourological Society; RSCS: Seoul National University Renal Stone Complexity Score; ROC: receiver operating characteristic; PCNL: percutaneous nephrolithotomy

The ROC curve was generated to analyze the probability of scoring tools for predicting effectiveness (Figure 1). All the curves were above the reference diagonal; hence, the test was interpretable. The interpretative details are presented in Table 4. Apart from the RSCS, which showed better sensitivity, the other scoring tools were similar in sensitivity and specificity for predicting effectiveness.

**Table 4:** Area under the curve (AUC), sensitivity, and specificity of STONE score, CROES nomogram, Guy’s Stone Score, and RSCS for predicting the effectiveness of multiple-tract mini-PCNL for renal stones STONE: Size of the stone, Topography or location, degree of Obstruction of the urinary system, Number of stones, and Evaluation of Hounsfield units; CROES: Clinical Research Office of the Endourological Society; RSCS: Seoul National University Renal Stone Complexity Score; PCNL: percutaneous nephrolithotomy

Scoring Tool	Area	Asymptotic Significance	Asymptotic 95% Confidence Interval		Curve coordinates cut-off	Sensitivity	Specificity
			Lower Bound	Upper Bound			
Guy's Stone Score	.766	.000	.677	.856	2	60.0%	56.8%
CROES nomogram	.858	.000	.790	.925	226	60.0%	49.5%
RSCS	.703	.002	.598	.807	4	86.7%	52.6%
STONE score	.799	.000	.711	.888	7	46.7%	55.8%

Contingency table analysis was used to assess the association between various scoring tools and outcomes, as shown in Table 5. A statistically significant association (p-value <0.05) was recorded for all scoring tools. Odds ratio with 95%CI was calculated to measure the strength of association, which was 7.093 (95%CI 2.40-20.89), 9.333 (95%CI 2.92-29.81), 11.70 (95%CI 2.56-53.38), and 3.450 (95%CI 1.25-9.53) for STONE score, GSS, CROES nomogram, and RSCS, respectively. The odds ratio was more than 1, hence statistically significant.

**Table 5:** Contingency table analysis and odds ratio for STONE score, CROES nomogram, Guy’s Stone Score, and RSCS for predicting the effectiveness of multiple tract mini-PCNL for renal stones STONE: Size of the stone, Topography or location, degree of Obstruction of the urinary system, Number of stones, and Evaluation of Hounsfield units; CROES: Clinical Research Office of the Endourological Society; RSCS: Seoul National University Renal Stone Complexity Score; PCNL: percutaneous nephrolithotomy

Scoring Tools		Stone-Free Status		p-value	Cramér's V value	Odds ratio (95%CI)
		Achieved (n = 86)	Not achieved (n = 24)			
STONE score, n (%)	≤7	56 (91.8%)	05 (8.2%)	<0.001	0.468	7.093 (2.40-20.89)
	>7	30 (61.2%)	19 (38.8%)			
Guy's Stone Score, n (%)	Grade 1&2	56 (93.3%)	04 (6.7%)	<0.001	0.402	9.333 (2.92-29.81)
	Grade 3&4	30 (60.0%)	20 (40.0%)			
CROES nomogram, n (%)	≤226	52 (96.3%)	02 (3.7%)	<0.001	0.519	11.70 (2.56-53.38)
	>226	32 (57.1%)	24 (42.9%)			
RSCS, n (%)	≤4	46 (88.5%)	06 (11.5%)	0.013	0.236	3.450 (1.25-9.53)
	>4	40 (69.0%)	18 (31.0%)			

## Discussion

PCNL has evolved into the norm in treating renal calculi. The procedure's efficacy depends on numerous determinants influencing the calculi's accessibility and ultimate removal. The aforementioned factors comprise the stone burden, quantity, structure, site of calculi, BMI, and aberrant renal architecture. A single substantial indicator of accomplishment, however, is unavailable. As a result, numerous authors constructed predictive models by incorporating such diverse metrics [6,10]. This study evaluated the performance of four key models designed to predict the effectiveness of multiple tract mini-PCNL.

Overall, SFS was observed in 78.2% (n=86) of patients. This percentage is slightly higher than the report of Yarimuglo et al., in which SFS was observed in 71.4% of patients [11]. This may be attributed to the fact that SFS was defined by the complete absence of stones on follow-up CT scan as compared to our study, where <4mm residual stones were also considered SFS. In order to enhance the success rate, numerous researchers assessed the efficacy of multitrack PCNL, whether used in conjunction with standard PCNL, mPCNL, or an analogous arrangement. Manohar et al. achieved complete stone clearance in 86% of instances using mPCNL with a multitrack approach [12]. Cho et al. discovered that multi-tract mPCNL remains reliable and efficient for adequately selected individuals [13]. Fei et al. reported 78% SFS with a similar approach. They found that multi-tract PCNL was indispensable for enhancing the overall extraction of stones alongside decreasing the demand for additional extracorporeal shockwave lithotripsy (ESWL) or second-look PCNL. Nevertheless, a flexible nephroscope was employed to extract minute peripheral fragments, verify the removal of stones, and determine the necessity and site for an additional incision [14].

In the current study, all four scoring tools (STONE score, GSS, CROES nomogram, RSCS) were similar in performance in predicting the outcomes. A universally acknowledged stone assessment system for forecasting the results following PCNL is yet to materialize, and there are inconsistencies among authors regarding the efficacy of stone-scoring tools in predicting outcomes [15]. Tailly et al. demonstrated that the predictive ability of SFS for STONE score, GSS, and CROES nomogram is similar to our findings [16]. Furthermore, it was documented by Bozkurt et al. that there exists a noteworthy correlation between the GSS and CROES nomogram in both SFS and complications [6]. Additionally, Noureldin et al. found that GSS and STONE score exhibited comparable associations with SFS; however, no statistically significant relationships with complications were observed [17].

A comparative analysis indicated that the STONE score could be employed for predicting SFS [18]. In instances involving structural aberrations, Kocaaslan et al. found that the CROES nomogram had a strong correlation with the effectiveness of PCNL. In contrast, other tools failed to forecast SFS and complications [19]. In comparison, Choi et al. determined that only GSS could predict SFS following PCNL [20]. In another analysis, Sfoungaristos et al. found that the STONE score was the sole predictor of the SFS, and others failed to do so [21]. The findings of the current study revealed that all four scoring tools were significantly correlated with SFS and predicted SFS to an equivalent degree.

Our study has limitations which include being a single-centre study and having a small sample size and a retrospective study design. However, despite these limitations, it is our belief that the study would provide a platform for future large-scale prospective studies on this topic.

## Conclusions

PCNL for retrieval of renal stones was 78.2%, and the complication rate was 13.6%, demonstrating that multi-tract mPCNL is an effective and safe tool for managing renal stones. The clinical utility of scoring systems, including STONE score, GSS, CROES nomogram, and RSCS, demonstrated that all scores adequately predicted the absence of stones following the procedure. Pre-operatively, stone scoring tools help forecast the outcome in multiple-tract mPCNL.
